# Lack of Pregraduate Teaching on the Associations between the Built Environment, Physical Activity and Health in Swiss Architecture and Urban Design Degree Programs

**DOI:** 10.3390/ijerph18010015

**Published:** 2020-12-22

**Authors:** Matthias Zedi, Bengt Kayser

**Affiliations:** Institute of Sport Sciences, University of Lausanne, 1015 Lausanne, Switzerland; zedi.matthias@bluewin.ch

**Keywords:** physical activity, health, epidemiology, education, pedagogy, urbanism, architecture

## Abstract

Background: Lack of physical activity (PA) is the fourth risk factor for all-cause mortality. Regular PA reduces noncommunicable disease (NCD) and mortality risk. The built environment (BE) is a determinant of spontaneous daily PA. Professionals who plan and build the BE therefore affect public health. We tested the hypothesis of a lack of formal pregraduate training about associations between the BE, PA and health in architecture, landscape architecture, and urban design academic degree programs (DPs) in Switzerland. Methods: We reached out to all DPs in Switzerland to ask if and how these associations are taught. For those declaring to teach the topic, the program syllabus and course material were inspected. Results and discussion: For 30 out of 33 identified programs, information for the analysis was obtained. A total of 18 declared teaching the BE, PA and health associations, but this could be confirmed for only 5 after verifying the course content. Teaching principles of building PA-promoting BE represents an underutilized potential for public health promotion. Conclusions: There is a need to introduce formal learning objectives in architecture, landscape architecture, and urban design DPs in Switzerland on the associations between BE, PA and health. It is likely that similar needs exist in other countries.

## 1. Introduction

Noncommunicable diseases (NCDs) such as cardiovascular diseases, diabetes, cancer, and chronic respiratory diseases are leading causes of mortality globally [1] as well as in Switzerland [2]. The lack of physical activity (PA) is a determinant of NCDs and leads to premature mortality [3,4].

The World Health Organization (WHO) recommends a minimum of 150 min/week of moderate intensity PA or 75 min/week of high intensity PA [5]. Yet, a quarter of the world’s population is not physically active enough and if current trends continue, the WHO goal of a 10% reduction in insufficient physical activity by 2025 will not be met [6]. Similarly to other affluent countries, 20–25% of adults in Switzerland do not meet these recommendations [7]. Physical inactivity also comes with a stiff direct cost amounting to 2.5 billion Swiss Francs every year, i.e., 290 Swiss Francs/person [8].

Everyday activities such as walking to a bus stop or a shop, or cycling to work, contribute to better health outcomes [9]. Hence, the BE influences our daily physical activity [10,11,12]. According to Handy [10] (p. 65) “the built environment (BE) comprises urban design, land use, the transportation system and encompasses patterns of human activity within the physical environment”. A more active design of the BE can help to reach population activity recommendations and reduce NCDs [13,14].

Most studies on the BE and PA focused on where people live. People also spend a lot of time inside buildings, for work, study, shopping or leisure. The site and design of buildings affect PA as well [15]. Elements such as access, stairs, spatial design, locker rooms and showers promote PA [15]. Stair climbing is an activity with a higher energy consumption than normal walking [16] and improves cardiovascular health, arterial blood pressure and leg strength [17,18,19,20]. Nicoll [21] reported that in ten academic buildings travel distance from the stairs to the nearest entrance, and elevator or staircase accessibility, were associated with stair use. An active building design can increase stair use and represents health-promoting potential [22]. Additionally, a PA-promoting office building can decrease workplace sitting time [23,24] and reduce lower-back pain [25].

Building a physical activity promoting BE represents an opportunity to increase people’s PA and health. However, to allow architects, landscape architects and urban planners to implement such active designs, they need to have learned about the rational, the knowledge and the knowhow to do so. Informal contacts with active professionals in the field and with actors in various Swiss degree programs (DPs) in architecture, landscape architecture and urban design led us to formulate the hypothesis of a lack of formal pregraduate training in these DPs about the associations between the BE, PA and health and the general principles of epidemiology and public health. To test this hypothesis and to find out what is taught about the associations between the BE, PA and health, we conducted a survey of all Swiss DPs providing pregraduate training in architecture, landscape architecture and urban design.

## 2. Materials and Methods

By means of online questionnaires and direct contacts we surveyed Swiss architecture, landscape architecture, and urban design DPs. The study protocol did not fall under the Swiss law on research on human subjects and formal ethical approval from the local institutional review board was waived (Supplementary Materials).

### 2.1. Academic Degree Programs in Switzerland

All existing relevant Swiss degree programs (DPs) were identified. Twelve academic institutions offer a bachelor DP in architecture. Six architecture DPs are offered at master level. The Universities of Bern, Geneva and Fribourg offer a joint master of architecture. Landscape architecture can be studied in two bachelor DPs at the university of applied sciences (UAS) in Geneva and Rapperswil, and in two master DPs, at the Swiss Federal Institute of Technology Zurich (ETHZ) and at the University of Applied Sciences (UAS) Rapperswil. The master at the UAS Rapperswil has two tracks, landscape architecture and spatial development. Although there is just one bachelor DP for urban design, at the UAS Rapperswil, there are five master DPs, at the Universities of Geneva and Lausanne, the ETHZ, and the UAS Rapperswil (double track master mentioned before). At the Swiss Federal Institute of Technology Lausanne (EPFL), there is a minor in territorial and urban design development. This interdisciplinary minor consists of a group of compulsory and optional courses offered by the faculty of Architecture, Civil and Environmental Engineering. This minor is open for all master students across the three sections of this faculty. Further education in spatial planning exists in the form of a Master of Advanced Studies (MAS), which is offered at five universities; the EPFL and the University of Geneva offer one of them jointly. Detailed information on all DPs can be found in the Supplemental Material.

### 2.2. Questionnaire

Requests to fill out an online questionnaire (in French and German) were sent by email to all of the DPs’ responsible persons. Contacts for DPs were found on the homepages of the academic institutions. Some contact persons transferred the request to someone with better knowledge of the DP content. Respondents were asked to enter their name, academic institution, function, and role in defining the DP content. They were then asked if there was a specific course in their program that discussed the links between BE, PA and health:

(1) “Does your university/college propose one or more lecture(s) in one or more course(s) that explicitly deal(s) with the relationships between the built environment, physical activity and health?”

Since it was possible that this link was taught, but among other learning objectives in another course, if the answer was no, they were asked if this topic was touched upon in a different course of study:

(2) “Is the connection between physical activity and the built environment dealt with in another lecture, but not as primary learning content?”

If the answer to questions 1 or 2 was yes, respondents were then asked to specify the name of the course, in which program it was taught, and if the syllabus of the course could be made available.

In the second part of the questionnaire respondents were asked if any course existed that taught health, epidemiology and public health in their program:

(3) “Does your university/college have a lecture in one or more courses that deals with health, epidemiology or public health?”

If the answer was no, the survey participants were asked if there was a course in which these topics were mentioned, but not as a part of the main course content:

(4) “Are health, epidemiological or public health topics dealt with in another lecture, but not as primary learning content?”

If the answer to one of these questions was yes, respondents were again asked to enter the name of the course, the DP in which the course was taught, and if the description of the course could be made available. For all of the identified courses, further information about the course contents was requested (see Section 2.4 Content Verification).

### 2.3. Data Collection

**Figure 1:**
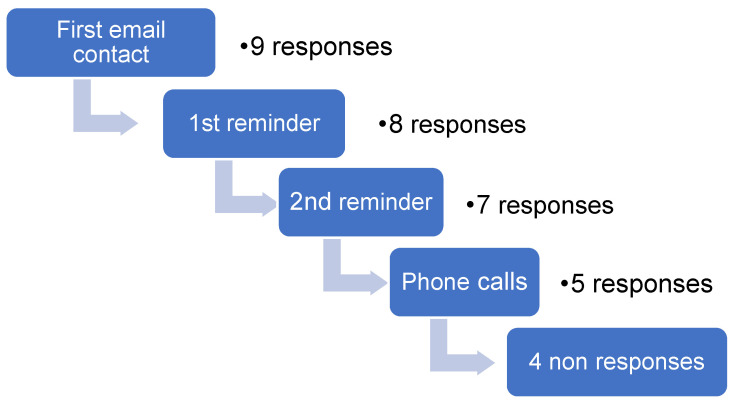
Flow diagram of recruitment.

After two email reminders, the non-responsive DPs were surveyed by phone and an investigator (MZ) directly filled out the questionnaire with the contact person (Figure 1). If no response was obtained, the study program descriptions of these DPs were analyzed, if available. For the non-responders, the program descriptions of the DP, available on the university websites, were screened for the following keywords: physical activity, activities, active, health, public health and epidemiology. If these keywords were mentioned, the description was marked for content verification and the responsible professor for the specific course content was directly contacted by e-mail or telephone for further information. The professor was asked to provide access to course material for content verification (see Section 2.4 Content Verification).

### 2.4. Content Verification and Analysis

When the respondent declared that there was a course that taught the relationship between BE, PA, and/or one on public health and epidemiology, the course descriptions and learning objectives were inspected. Since it was possible that a course taught the topic, but not as one of its main objectives, course descriptions were analyzed for mentions of health and PA. The responses given in the questionnaires and by telephone were compared to the actual content of the documents. To fully analyze the content of the courses, further information was requested from course directors and professors teaching the courses. They were contacted by email or telephone, and asked to confirm that not only the association between BE and PA is taught, but also the positive effects of PA on health, as well as the links to epidemiology and public health. For the verification, they were asked to provide, if possible, the course material (course syllabus, slides) that could prove that these relations were actually taught to the students. The received material was then inspected for verification. The verification was successful if, on at least one slide or in one document, the health benefits of PA in connection to the BE were described in any form. If no course material could be made available, an explicit and written confirmation from the professor teaching the subject was accepted as proof in the analysis.

## 3. Results

Responses were obtained from 29 of the 33 DPs contacted (Figure 1)

The data was provided by 16 program directors, 5 professors teaching relevant courses, 3 study coordinators, 3 administrators, 1 study advisor and 1 course co-director. Out of the 29 respondents, 25 declared that they have an active role in designing the DPs, 3 in coordinating the DPs, while one respondent did not actively participate in the design of the DPs. For one masters program, in spatial development and infrastructure systems at the ETHZ, no response could be obtained. After screening the program description online, a course was identified which mentioned a link between BE and PA. Through direct contact with the professor responsible for this course, information about the course could then be obtained and inspected.

### 3.1. The Relations between the BE, PA and Health

Seven DPs declared the inclusion of a specific course teaching the relations between the BE, PA and health, while eleven DPs said the subject was taught along other concepts in another course of the DP. Twelve DPs answered negatively. Subdividing, none of the 17 architecture DPs declared a specific course on the relations between the BE, PA and health, while 5 architecture DPs declared some limited teaching on the topic. Three landscape architecture DPs declared specific courses, one some limited teaching. In urban design, five declared specific courses, five some limited teaching (see supplemental online information for a more detailed analysis for each DP category). The Master in Spatial Development and Landscape Architecture is listed twice—once in landscape architecture and once in urbanism, because of the two tracks.

### 3.2. Content Verification

**Figure 2:**
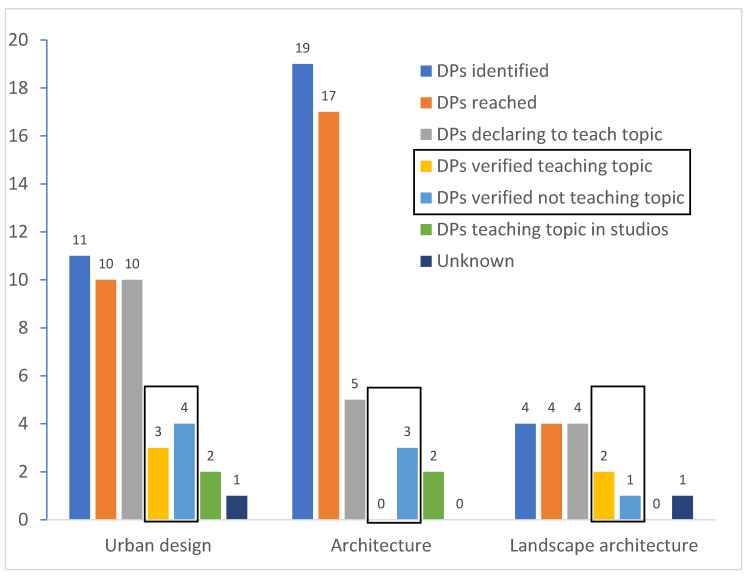
Summary of the results. A Master in Spatial Development and Landscape Architecture is listed twice—once in landscape architecture and once in urbanism, because of the two separate study tracks. Boxes indicate results after the verification of courses for actual topic content. Unknown indicates two courses that declared teaching the topic, but for which the actual course content could not be inspected.

Eighteen DPs declared that the relations between the BE, PA and health were present in at least one of the courses, or that courses existed with specific focus on the topic. However, after checking the syllabi or other documents of these courses to see if not only the relation between PA and the BE was taught, but also its positive effect on health (such as mitigating NCDs), this could be confirmed for only 5 out of 18 DPs. Four DPs declared that the subject is taught in so-called studios (i.e., project-oriented workshops) for which there was no course material available, and therefore it could not be directly confirmed whether it was formally taught. For two DPs no confirmation could be obtained. Upon verification, in seven DPs the BE-PA-health links were not taught, contrary to the declarations by the respondents (Figure 2).

None of the architecture DPs contained a course where the topic was specifically taught. Either the link between the BE and PA was only cursory explained without considering the health effects, or a link between the BE and health was taught without taking into account PA. For the five architecture DPs for which the respondents indicated that this link was taught, it could not be confirmed. For the two DPs at the University of Bern, it was declared that the subject was taught in design studios, but again this could not be confirmed.

For the landscape architecture DPs, all respondents indicated that the link between the BE, PA and health was taught. It could only be formally confirmed for the bachelor DP at UAS Rapperswil and the master DP at the ETHZ. In the master DP at UAS Rapperswil, the link was only cursorily taught. For the DP at the University of Geneva, no confirmation could be obtained.

For the urban design DPs, similar findings were obtained. All respondents of the ten DPs answered either question one or two of the questionnaire positively, indicating that the topic was taught in some way. In the Master in Environmental Sciences (MUSE) at the University of Geneva, and the Master of advanced studies in Spatial Development at UAS Rapperswil, it could be confirmed that there was a course which specifically focused on the topic. In the DPs at the University of Lausanne, it was briefly mentioned but was not a focus. For one DP, no confirmation could be obtained, in three it was not taught and for two DPs it was declared that the link was taught in design studios.

### 3.3. Health, Epidemiology and Public Health

In questions three and four of the questionnaire, the respondents were asked if in any of the respondent’s university programs there was a course that discussed concepts of health, epidemiology and public health. Three DPs indicated that a course treating health, epidemiology or public health existed in their core curriculum: (1) ETHZ, landscape architecture: “New city landscapes and health”; (2) University of Geneva, urban design: “Environment and health”; and (3) EPFL, urban design: “Exploratory data analysis and geovisualization”. Although the other 26 DPs did not include a course on public health or epidemiology in their core curriculum, there were numerous courses in other DPs at the same academic institutions that did teach one or the other of those subjects.

## 4. Discussion

We inventoried what is taught in general about health, epidemiology and public health, and in specific about the relationships between the BE, PA and health in Swiss pregraduate degrees in architecture, landscape architecture and urban design. Our main finding is an important overall lack of learning objectives and formal teaching content on these topics, with some rare and rather partial exceptions. Given the state of science in this field and its articulation with the UN’s sustainable development goals, this finding is disconcerting [26]. It is now well described how the BE can promote PA [13,14,27] and professionals in these disciplines therefore have an important impact on public health and the 2025 global physical activity target set by the WHO [6]. Already “in the 19th and early 20th centuries, architects and urban reformers in New York City and elsewhere helped defeat infectious diseases like cholera and tuberculosis by improving buildings, streets, neighborhoods, clean water systems, and parks. In the 21st century, designers can again play a crucial role in combating the biggest public health epidemics of our time: obesity and related chronic diseases such as diabetes, heart disease, and some cancers” [28] (p. 12). In Switzerland, 77% of the population—or in absolute numbers, around 6.5 million people—now live in growing urban areas [29]. City planners should therefore actively “consider nature and landscape quality as a contributor to structural health promotion and enhance this quality by means of revaluation measures” [30] (p. 27). The inclusion of this knowledge and knowhow in Swiss DPs which teach future professionals about such environmental planning is therefore mandatory [31]. Although several of the surveyed DPs declared that they included some course material on the health effects of PA in their program, to date only two urban design courses and one landscape architecture DP provided verified formal content focusing on the relation between PA and health. A positive trend was observed for a newly created master at the ETHZ, where this teaching content was announced for 2021. To understand why a movement-friendly environment is so important for the health of society, the influence of PA on health should be formally taught to all students in such DPs.

While in most of the landscape architecture and urban design DPs the topic was covered, at least partially, none of the architecture DPs taught the relations between the BE, PA and health in any comprehensive way. Only five out of seventeen DPs declared having courses in which this relation was taught. However, upon verification of course content, even if PA was mentioned in a course, the link to the health benefits of PA was never made explicit. The courses at the ETHZ in which PA was mentioned were courses about landscape architecture, but did not address promoting PA inside buildings. Design studios were reported to mention this relation, but as the projects of these studios change every year, and because not all students take the same design studios, they are not part of the core curriculum. Other courses indicating a mention of the importance of BE on health in general tended to focus on health parameters like ergonomic furniture, air quality or chemicals, but not PA. According to Klepeis et al. [32], most people in the USA spend 90% of their time inside and depend a lot on the way their indoor environment is designed [33]. In Switzerland this may be somewhat less, but the point remains that peoples’ PA behavior is influenced both outside and inside by the BE.

We propose that the core curriculum of any DP in architecture, landscape architecture and urban design includes learning objectives and formal teaching material on these themes, preparing the students for their future professions. Ad minima, this core curriculum could contain a historical perspective, the current inactivity epidemic and its health consequences, the relationship between PA and NCDs, the influence of the BE on PA, BE design principles promoting PA and showcases of examples of active design. Based on a model curriculum developed by Botchwey et al. [34] and other studies [35], we herein propose minimal content for seven themes with possible session topics of such a core curriculum (see Table 1).

The relationships between the BE, PA and health, especially inside buildings, are not yet fully understood [33,36]. Nevertheless, the potential to improve the conditions for a healthy life and to increase health by planning movement-friendly environments is large globally [31] as well as in Switzerland [30]. According to the Swiss Federal Office of Public Health [30], this potential can “only be utilized if all policy areas make an additional contribution to health in the years ahead. In the interest of policy coherence, health concerns must be proactively incorporated in sectoral policies. A more comprehensive and coherent approach to policy-making is required at a federal level in order to exploit synergies and to support the development of a comprehensive health policy” [30] (p. 25). Such synergies need to be orchestrated at higher education institutions where complementary expertise can be brought together. At two thirds of the academic institutions of the analyzed DPs, courses about health, public health or epidemiology exist in other departments such as medicine and health science. Collaboration with those departments must be encouraged, bi-directionally: both health and BE professionals need to learn about the effects of the BE on health. Importantly, most BE measures that promote PA also help in reaching the UN’s sustainability goals [26].

### Limitations

Those responsible for the DPs were asked to identify courses where the relations between the BE, PA and health are taught. However, no precise definition was given for PA. This led to some confusion, as PA was sometimes equated with exercise or sport. An additional limitation was that the respondents did not always exactly know what their colleagues taught in their courses and could not always provide detailed information about the actual course content. For this reason, some courses that might have mentioned the investigated subject being an aside may have slipped through our analysis. For those courses, where no further course content was received, the only way to find out if this subject was taught, would have been to attend the courses in person. Nevertheless, it was clear that for those DPs there were no clear indications for formal learning objectives and content in the program. For architecture and landscape architecture, only bachelor and master DPs were analyzed, whereas for urban design a postgraduate master of advanced studies was added to the analysis. It cannot be ignored that there are such postgraduate master and doctoral programs in other fields where the topic may attract more attention than in the pregraduate bachelor and master DPs.

## 5. Conclusions

It is known that the BE influences our PA behavior and therefore has an impact on the leading cause of mortality—NCDs. The promotion of movement-friendly environments is a promising application area for the promotion of PA and health. Nevertheless, it is rarely taught in the Swiss architecture, landscape architecture and urban design DPs. Out of 30 analyzed DPs, only five formally and fully taught the relations between the BE, PA and health. In none of the 17 architecture DPs could it be confirmed that students are made aware of the effect our BE has on PA and on health. In contrast, two out of the four landscape architecture DPs taught the importance of promoting PA when planning environments. In urban planning, the importance of PA in our society has indeed become a part of some urban planning degrees. However, in the courses, the focus is mainly on the technical part, on how to provide infrastructure that promotes PA, without considering the health aspects. This aspect is only fully developed in 3 out of 10 urbanism DPs. These findings indicate that a lot of students of all three formations were not made aware of the consequences physical inactivity has for our health, and how in their future professions they can have an impact on our health by planning movement-friendly environments. An interdisciplinary cooperation between physicians, sport scientists, epidemiologists, sociologists and planning professionals needs to be encouraged to overcome this lack of knowledge. In conclusion, there is a need to introduce formal learning objectives in architecture, landscape architecture, and urban design DPs in Switzerland on the associations between BE, PA and health. It is likely that similar needs exist in other countries. We recommend repetition of our study in other countries combined with active advocacy for the formal introduction of the core curriculum we herein propose.

## Figures and Tables

**Table 1:** A proposal for a core curriculum.

Theme	Session Topics
**Foundational Knowledge**	
Epidemiology	Public health and planning history.
	Historical and current theories on the relationship between the built environment and public health.
	Inactivity pandemic.
Spatial epidemiology and health	Non-communicable diseases (NCDs).
	Physical activity relationship with health and built environment.
	Evidence for the built environment–health link.
	Financial cost of inactivity.
**Application**	
Interdisciplinarity	Benefits of and barriers to working in an interdisciplinary environment.
	Studies and methodologies developed by sociologists, anthropologists, urban planners, sport scientists, epidemiologists and architects to evaluate the health impacts of the built environment.
	Co-benefits of healthy environments in terms of sustainability and economy.
Designing the built environment for health	Analysis of best practices.
	Tools and techniques available to connect urban planning and public health.
	Develop and implement new programs and policies that utilize built environment and design to promote public health.
Monitoring	Methods used to assess the built environment and its impact on health.
	Assessing the population’s needs.
**Human Dimension**	
Communication	Describe the options available to promote healthy design decisions.
	Present ideas linking evidence with policy guidance to local agency representatives.
Awareness	Raising awareness about health behavior of an individual’s everyday life.

## Data Availability

Data can be made available upon reasonable request to the corresponding author.

## Supplementary Materials

The following is available online at https://www.mdpi.com/1660-4601/18/1/15/s1, Swiss degree program content.
