# Supplementary material for: Lack of Pregraduate Teaching on the Associations between the Built Environment, Physical Activity and Health in Swiss Architecture and Urban Design Degree Programs
Source: Int J Environ Res Public Health. 2020 Dec 22;18(1):15. doi: 10.3390/ijerph18010015 (PMC7792804; doi:10.3390/ijerph18010015)
Supplement: Supplementary file 1 [file ijerph-18-00015-s001.pdf]

Supplemental online information: Swiss degree program content

1. Architecture

According to respondents of the questionnaire, the relationships between BE, PA and health was taught in three bachelor and two master programs in architecture, but no course existed which focused solely on the thematic. Hereafter, the listed courses which teach the investigated relationship are described and analyzed in more detail.

ETHZ

At the ETHZ there were courses that only briefly mentioned the relation of BE and PA to the students. During the 2019 spring semester, the course "Architectural Design V-IX: The Dark Side of the Sihl" (a river in Zurich), mentioned the influence of BE on PA in the course description. The objective of the course was to "develop a visionary landscape project that strengthens the relationship between the city, its inhabitants and the dark side of the Sihl". "The goal of this studio is to create a new landscape topology that will enable a stronger and more active relationship and access to the Sihl for the local population. The stretch of river located at the center of town offers great landscape potential for promenade, leisure and bathing. It has been ignored over the past years for various reasons, least of which being the threat of flooding". According to the professor, this course held the assumption that the promotion of sport and leisure activities leads to an improvement of health in general. However, the effects of PA on health were not specifically discussed, and although it was mentioned in the course description that the goal of the course was to create a more active environment, the effect of BE on PA and the thereby resulting health benefits were not specifically mentioned in the course. In another architectural design course, 'The alps as common ground – Marseille,' the investigated relationship was partially taught, but in the course

description it was not specifically mentioned. These courses are taught in the bachelor and master of Architecture at ETHZ. In the bachelor two additional courses were found. According to the responses to the questionnaire, the courses 'Landscape architecture 1' and 'Landscape architecture 2' discussed the relationships between BE, PA and health. While no evidence of this was found in the course descriptions, the descriptions were very general so this does not necessarily mean that the relationship was not discussed. One description read "rather than concentrating only on questions of style, the series will also tackle issues such as revitalization, sustainability etc." According to the objective, an "overview to contemporary and forthcoming tasks of landscape architecture" was given in the course. Although the subject of a movement-friendly environment would fit into these courses, the relation between PA and health were at best briefly mentioned as an aside in these courses.

UAS Northwestern Switzerland

At the University of Applied Sciences and Arts Northwestern Switzerland, two courses teaching the links between BE, PA and health were listed for the bachelor's degree. In the courses '*Social Science 1*' and '*Social Science 2*' students should be made aware of the fact that the BE influences our daily PA and health. The course '*Social Science 1*' dealt with public spaces in an urban context with changing focus based on a current issue while 'Social Science 2' did this in a suburban context. However, in the course descriptions of these two courses, no specific evidence could be found for the influence of BE on PA. Although these two courses were listed by the DP responsible, according to the professor the effect of the BE on PA was not taught, nor the relation between PA and health.

UAS Bern

At the UAS Bern, students do not learn that the BE can affect one's PA behavior in any course, but in different design studios. According to the respondents of the questionnaire, faculty includes this topic when discussing specific student design projects. For the design studios no descriptions or further information was available, and can therefore it could not be checked. The same was the case for the joint master's DP.

2. Landscape architecture

In three landscape architecture DPs there were courses focusing on the relation between BE, PA and health. In one DP it was only part of other courses, which had other main focuses. See the detailed analysis of the courses below.

UAS Rapperswil

In the bachelor's DP three main modules claimed to include the relation between the BE, PA and health: "Design", "Landscape development", and "Open space planning". Six "Design" modules were offered during the Bachelor, three "Landscape development" modules, and seven "Open space planning" modules. None of these course descriptions explicitly mentioned the relationships. The course description of "Design 4" stated that "the environmental design of a housing estate with questions on sustainable development and a holistic design with special consideration of user behavior" is taught during the course. In the course description for "Design 6," it says that students learn to "cooperate in an interdisciplinary team during the planification of projects". According to the descriptions, all three of the "landscape development" courses include the "analysis and

evaluation of the cultural, natural and recreational landscape”. In “Free space planning 1,” one of the aims is to teach “knowledge about current issues like densification, sustainability and the quality of living space”. According to the responsible professor, an invited expert in the field of active mobility and health has taught in this course since 2019. The expert, the former project coordinator of “Moving municipalities”, teaches the course “Active mobility and health” as part of the “Open space planning” module. In this course she defines health and the factors which influence it. Also, the “Epidemic of inactivity” is explained in her course, and how PA can be used to face this challenge. Students are also taught how BE can be created to promote health. Some ‘best practices’ are shown at the end of her lecture. By contrast, according to the responses to the questionnaire, the master’s DP in landscape architecture does not mention the BE-PA-health-relation in any course.

Geneva School of Engineering, Architecture and Landscape

At the School of engineering, architecture and landscape Geneva, the relation between BE, PA or health was listed in three different modules. In the first module, “Applied techniques”, this relation was present in two different courses. The first one takes into account the “persons with reduced mobility and public space” and the second “non-motorized mobility”. A course description was only available for the module in general and not for these specific courses. In the description of the applied “techniques” module, no evidence for the investigated link could be found. The second module, “Sociology of public spaces”, did not specifically mention this link in the description either, but as the objectives of the course were “to identify the social and historic characteristics of a given territory” and to “analyze the different requests in the project approach,” the link could be inferred. The third module was an optional module and, according to the respondents, contained three courses that discuss the PA-BE-relationship. In “Playground and nature”, “Urban agriculture / local problems of nutrition”, and “Mobility”, these relationships were brought to students’ attention.

However, no course description was available for the optional module. The relationship between BE and PA seemed present in this DP, but it could not be confirmed whether or not the students were made aware of the positive effects of PA on health, because no further information could be obtained.

ETHZ

In the master's DP at ETHZ there were two courses which focused on the relation between BE, PA and health. The course "New cityscapes and health", already in the title, acknowledged a connection between BE and health. This relation was also taught in the second course, "Basics studio 2", as a main objective. As this master's DP was new (both courses planned for 2021) no course descriptions existed yet. However, the study regulations described the "Basic studio 2" and mentioned that "Basic studio 2" is a design studio that considers existing constructions as well as social, health, political, and environmental strategic issues. Since the consequence of inactivity is a politicized health subject, it is part of this design studio. According to the course director, health and PA issues are now integral part of the new Landscape Architecture Master at ETH Zurich.

3. Urban design

Five out of the ten urban design DPs declared to focus on the relation between BE, PA and health in at least one of the offered courses. In the other five DPs it was only an aside of courses focusing on other topics.

UAS Rapperswil

In the bachelor's DP at UAS Rapperswil, one course specifically focuses on the relation between BE and PA. In the module "Building the city 7", one course called "Movement-friendly residential areas" familiarized the students with the subject. "City development projects with a changing thematic focus" were part of this module, and, depending on the year, the thematic focus was the relation between BE and PA. However, a consequent link between PA and health was not made in any course during the bachelor's DP. When this relation was present, it was more of a side note. In the master's DP "Spatial development and landscape architecture", there was no course that specifically taught this link. However, in several modules, like "Building the city 2 - free space planning", and "Building the city 3", it was indeed mentioned. Two examples of courses where it was mentioned were "Recreational space" and "Sport and place". In traffic planning courses walking and cycling is part of the course content, and here the link between BE and PA is made as well. But again, the effect of PA on health is not specifically taught. In the MAS in spatial development, as a part of the CAS "Walking and cycling", there is a module focusing on movement and health. No course description or objectives were available, but the title, "Walking, cycling and health", suggests that the influence of PA on health is taught. The responsible professor provided insight into the course program of this module. One workshop is called "Movement promotion in daily life", and another "Movement-friendly living environment". Although no detailed course descriptions were available, the title and the course schedule showed that measures to promote walking and cycling are taught during this course, and the importance for public health is explained.

Although the relationship between BE and PA is a subject present in urban design DPs at Rapperswil, only during the CAS "Walking and cycling" section, the effects of movement-friendly environments on health are discussed.

University of Geneva

In the Master of Territorial Development (MDT) there was no course focusing on the BE and its influence on PA. However, according to the respondent in nearly every studio this relation has become a dimension of the projects. Even though in the description of the studios, the PA-concept is not formally present, in the “Interdisciplinarity studio”, for example, students are asked “to tackle a problem which has social dimensions in its globality”. One of these problems dealt with quality of life and responses to health challenges. In this studio an interdisciplinary approach was required. According to the respondent, the topic of health in combination with BE was not specifically taught, but was always considered when planning projects. Additionally, urban design experts from different domains, such as the promotion of PA and health, give invited lessons during these studios.

In the Master of Environmental Science (MUSE), the course “Environment and health” specifically taught the thematic. According to the description of the course, “the integration of the environment and health for the improvement of the quality of urban life is explained”. The course was based on the WHO ‘Healthy-Cities program’. Although no specific link to PA was made in the course description, the influence of inactivity on health was mentioned in the course and, according to the responsible professor, students get familiarized with how environments can be adapted for the promotion of PA. A specific course called “PA, health and BE” discusses the link between inactivity and NCDs, the recommendations for daily PA, and the effect of the BE on our lifestyle. The course pointed out a variety of possible solutions to mitigate these problems. Another course, which discussed the effect of the BE on PA and health, focused on walking and its promotion. It mentioned that regular walking prevents NCDs.

In the common MAS in Urban Design of the University of Geneva and the EPF Lausanne, there was no course that specifically taught the relation between BE and health. However, as in the Master of Territorial Development (MDT), this relation was taught in studios where students work on actual

projects. In addition to the studios, in one of the CAS sections, "Urban design and spatial planification", the association between BE and health was mentioned in some courses.

At the University of Geneva, the Master of Environmental Science was the only program that addressed the thematic in a specific course. In the Master of Territorial Development, and in the MAS in Urban Design, it was only mentioned in studios or without explicitly considering the relation between PA and health.

University of Lausanne

During the master's DP at the University of Lausanne, the course "Sustainable mobility: practices, planning and strategies" focuses on the link between the BE and PA. The description mentioned that the course focuses on "urban mobility that is less based on individual motorized transportation, such as cycling". This course description stated "mobility, shaped by the functioning of the economic system, by lifestyles and by spatial structures, increasingly intense and complex". Mobility "raises many issues in urban areas, like for example its impact on the quality of life". Furthermore, the course pointed out that "on this basis, numerous local authorities promote an urban mobility that is less based on motorized transportation, but pays more attention to active mobility, like cycling". The fact that cycling has a positive influence on health is not mentioned in the description, but in the introduction course the problem of NCDs, the resulting health cost, the need for the promotion of PA, and the influence of the BE on it is briefly described. The investigated subject was only mentioned on a few slides, but this means that students were at least made aware of these problems.

ETHZ

No responses from those contact person for the ETHZ DPs could be obtained. However, while analyzing all the course descriptions online, one course was found which focused on the investigated relationship. The course “Walking and cycling traffic” explained the “basics of the pedestrian traffic planning, as well as the planning of the infrastructure for the bicycle traffic”. According to the course description, students “acquire the basic knowledge in the field of pedestrian and bicycle traffic planning”. The characteristics of walking- and cycling-friendly infrastructure and the necessary techniques to implement these concepts into real planning are taught in this course. But according to the responsible professor, no link between PA and health is explicitly taught in the course. The focus is rather put on how to build the infrastructure. That the BE can bring health benefits is not mentioned in this master.

EPFL

In the minor “Territorial and urban design development,” two courses were listed that taught the relation between BE and PA. The courses “Health and environment” and “Health at work” are part of the module “Workplace environments & occupational risk”. Although the module explained the relationship between the quality of the working environment on public health, the link between PA and the BE was not listed in the description. The examples of what influences health at the workplace given in the description, such as chemical pollutants or noise, did not mention PA. According to the professor, only the environmental risks related to physical, chemical and biological agents were discussed in the course. PA is not a part of the discussed subjects. The relation between the BE, PA and health was not addressed in this minor either.

UAS Lucerne

In the MAS “Communal-, city- and regional development” at Lucerne no course focused on the relation between BE, PA and health. But in the CAS “Municipal and city development in transition”, the relation of BE, PA and health was part of the study content. The subject “Healthy municipalities,” and the promotion of health through the planning and enhancement of public spaces, for example, were a part of this CAS. According to the respondent, this subject could also be a topic analyzed in a master's thesis. The relation between the BE and health, therefore, was present in this program. But no course description and no further information was available to prove if the relation between PA and health was explicitly taught.
